# Safety and efficacy of remimazolam versus propofol sedation in gynecological procedures: a meta-analysis of East Asian randomized trials

**DOI:** 10.3389/fmed.2025.1701785

**Published:** 2025-11-26

**Authors:** Ou Jin, Wanqi Shao, Juan Lai, XiaoMin Yang

**Affiliations:** 1 Jiaxing Hospital of Traditional Chinese Medicine, Jiaxing, China; 2 Zhejiang Chinese Medical University Affiliated Jiaxing TCM Hospital, Jiaxing, Zhejiang, China

**Keywords:** sedation, hysteroscopy, remimazolam, propofol, gynecological surgery, anesthesia

## Abstract

**Background and Aim:**

Hysteroscopy necessitates appropriate sedation to ensure patient comfort and operative success. The relative safety profile of remimazolam compared to propofol in this context is unclear. This study evaluates the safety of remimazolam in comparison to propofol for sedation during hysteroscopy and other gynecological procedures.

**Methods:**

We systematically searched MEDLINE (PubMed), Embase, and Scopus from inception until September 2024. We included randomized controlled trials (RCTs) that compared remimazolam and propofol for sedation in hysteroscopy procedures. The analyses were conducted using a random-effects model by PRISMA guidelines. The main outcome was the incidence of total adverse events. Secondary outcomes comprised respiratory depression, hypotension, bradycardia, emergence time, and recovery time.

**Results:**

Thirteen RCTs comprising 1765 patients (remimazolam: *n* = 1,026; propofol: *n* = 739) met the inclusion criteria. The overall incidence of adverse events was significantly lower with remimazolam compared to propofol. Remimazolam was associated with lower risks of respiratory depression (OR, 0.25; 95% CI, 0.17–0.39; *p*  0.00001) and hypotension (OR, 0.30; 95% CI: 0.21–0.42; *p*  0.00001). No significant difference was observed in bradycardia (OR, 0.53; 95% CI, 0.28–1.02; *p* = 0.06). Recovery time [mean difference (MD), 0.18 min; 95% CI, −0.3, 0.65] and operation time (MD, 0.02 min; 95% CI, −1.0, 1.03) were almost similar for both groups.

**Conclusion:**

In patients undergoing gynecological procedures, remimazolam demonstrated a superior safety profile compared to propofol, with significantly lower rates of overall adverse events, respiratory depression, and hypotension. More studies are required to confirm these results.

**Systematic Review registration:**

https://www.crd.york.ac.uk/PROSPERO/, identifier CRD42024614416

## Introduction

Gynecological procedures, particularly hysteroscopy, are commonly performed and require adequate sedation to ensure patient comfort and facilitate the successful completion of the procedure (1, 2). For many years, propofol has been the most commonly used sedative due to its rapid onset and quick recovery characteristics, making it exceptionally appropriate for brief procedures (3, 4). However, despite its prevalent application, propofol possesses considerable limitations, including a restricted therapeutic period and the risk of adverse effects such as respiratory depression and hemodynamic instability (5–7). The identified hazards have prompted the pursuit of safer and more dependable alternatives for procedural sedation in hysteroscopy (7).

In recent years, remimazolam, a novel ultra-short-acting benzodiazepine, has emerged as a promising candidate for procedural sedation. Its unique pharmacological properties distinguish it from traditional sedatives like propofol (8–11). Notably, remimazolam undergoes organ-independent metabolism, which reduces the risk of accumulation and prolonged sedation, particularly in patients with hepatic or renal impairment (11, 12). Additionally, the availability of flumazenil which is a specific antagonist, allows for rapid reversal of sedation in the event of complications, further enhancing its safety profile (13). These characteristics suggest that remimazolam may offer significant advantages over propofol, particularly in terms of reducing the incidence of adverse events (11).

Despite these potential benefits, there is a notable lack of comprehensive evidence comparing the safety profiles of remimazolam and propofol specifically in the context of hysteroscopy. While previous meta-analyses have explored the efficacy and safety of remimazolam in various procedural settings, none have focused exclusively on hysteroscopy or provided a detailed analysis of adverse events associated with its use in this specific procedure (5, 14, 15). This gap in the literature highlights the need for a targeted evaluation of the safety of remimazolam relative to propofol in gynecological procedures.

We conducted this systematic review and meta-analysis to evaluate the safety of remimazolam versus propofol during gynecological procedures.

## Methods

This systematic review and meta-analysis adhered to the Preferred Reporting Items for Systematic Reviews and Meta-Analyses (PRISMA) guidelines (16) and is registered with PROSPERO (CRD42024614416).

### Data sources and searches

We searched multiple databases, MEDLINE (Pubmed), Embase, Scopus, and Web of Science, to collect studies that were relevant. This search spanned from the inception of these databases up to 24th of September 2024. We developed a search strategy incorporating terms related to “remimazolam,” “propofol,” and “hysteroscopy,” using different combinations and words (Supple search strategy). Importantly, we did not impose any language restrictions during our searches to ensure a broad inclusion of studies.

### Study selection

The selection of studies was performed independently by two investigators who screened titles, abstracts, and full texts. We established specific inclusion criteria to ensure that only relevant studies were considered. The inclusion criteria was as per PICOS framework.

P (Population): Studies focused on sedation during gynecological procedures.

I (Intervention): Studies comparing remimazolam to propofol.

C (Comparison): Studies comparing remimazolam to propofol.

O (Outcomes): Studies that included data on main outcomes, such as adverse events or other important outcomes of interest.

S (Study Design): Randomized controlled trials (RCTs).

Conversely, we excluded studies based on the following criteria:

1. Non-randomized studies.
2. Case reports or only abstract.
3. Duplicate publications.
4. Studies lacking sufficient data reporting, ie, not reporting adverse events or other important outcomes of interest.

### Data extraction

Data extraction was carried out independently by two reviewers using standardized forms designed to capture essential information. The extracted data included study characteristics, patient demographics, details of the interventions, and relevant outcomes. In case disagreements arose, we resolved them through consulting a third reviewer.

### Outcomes and definitions

The primary outcome of our meta-analysis was the overall incidence of adverse events (AEs) (defined as any type of adverse event or adverse effect that occurred during the procedure). We also evaluated several secondary outcomes, including specific adverse events such as respiratory depression (defined as an oxygen saturation level below 90% or a respiratory rate of fewer than eight breaths per minute), hypotension (defined as a systolic blood pressure below 90 mmHg or a mean arterial pressure below 65 mmHg), and bradycardia (defined as a heart rate of fewer than 50 beats per minute), as well as recovery parameters like emergence time (defined as time needed for patient to wake up) and recovery time (defined as the total time needed for the patient to return to a normal state), which includes the time from the initial drug administration to discharge from the Post-Anesthesia Care Unit (PACU).

### Statistical analysis

We conducted this meta-analysis using Review Manager 5.4, a tool provided by The Cochrane Collaboration. To analyze dichotomous outcomes, we calculated odd ratios (ORs) along with 95% confidence intervals (95% CIs) using the Mantel–Haenszel method. For continuous outcomes, we calculated mean differences (MDs) and their corresponding 95% CIs using the inverse variance method. Given the anticipated clinical heterogeneity among the included studies, we employed random-effects models. To evaluate the degree of statistical heterogeneity, we used *I*^2^ statistics, with thresholds of 25%–49%, 50%–74%, and over 75% indicating low, moderate, and high heterogeneity, respectively (17). A *p*-value of 0.05 or less was considered significant.

### Risk of Bias assessment

To assess the quality of the RCTs in our analysis, we utilized the Cochrane Collaboration’s risk of bias assessment tool. Each study was rated for risk of bias as “low” (minimal risk), “high” (significant risk), or “some concerns” (uncertainty due to incomplete information). We examined factors such as the randomization of participant assignment to treatment groups, the potential impact of missing data on results, the evaluation of outcomes, and adherence to the original study plan (18).

### Publication bias

To assess publication bias, we generated funnel plots and performed Egger’s test, applicable when 10 or more studies were available for analysis. Additionally, we conducted sensitivity analyses to evaluate the robustness of our results. We systematically removed each study from our analysis to observe its impact on the outcome. When a study that demonstrated a significant influence on the results was excluded, we conducted further analysis to assess the implications of this removal (19).

## Results

### Study selection and characteristics

**Figure 1:**
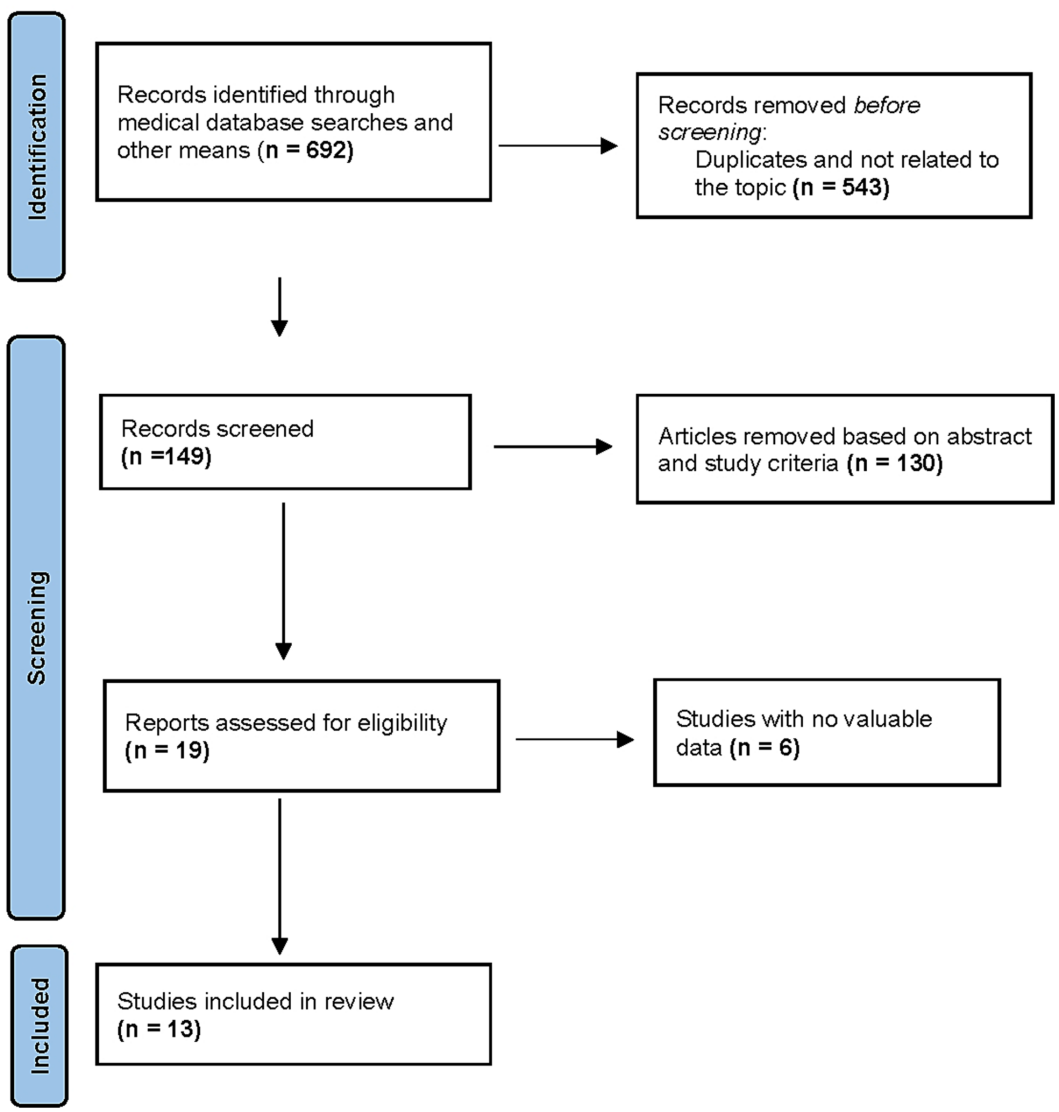
PRISMA flow diagram of study selection.

The initial search identified 692 records. After removing duplicates, 149 studies were selected. After screening titles and abstracts, 130 studies were removed, and19 full-text articles were assessed for eligibility. Thirteen RCTs met inclusion criteria, comprising 1,765 patients (remimazolam: *n* = 1,026; propofol: *n* = 739) (6, 7, 20–30) (Figure 1). Study characteristics are summarized in Tables 1 and 2. Sample sizes ranged from 8 to 125 patients in each group. All studies were conducted in Asia [12 in China (6, 7, 20, 22–30) and one in Japan (21)] between 2021 and 2024. The participants’ mean age ranged between 30 and 71 years and mean BMI ranged from 20.7 to 24.7. Four studies were conducted as multiple-arm investigations (6, 20, 28, 29), comparing various doses of remimazolam to a propofol group independently. Six studies utilized remimazolam tosylate (6, 25, 26, 28–30), while seven studies utilized remimazolam besylate (7, 20–24, 29). Nine studies focused on the hysteroscopy procedure, while two investigated different gynecological surgical procedures, and one each included cervical conization and surgical abortion. One study was multi-center, and the other 12 studies were single-center. One study was conducted as a multicenter study, whereas the other 12 studies were conducted at a single center.

**Table 1:** Characteristics of included studies.

Study	Design	Patients	Age	BMI	Procedure	Dosage/Kg
Zhang et al. (6) China	Single center	RB = 41	43.8 ± 8.0	24.7 ± 2.7	Hysteroscopy	0.2 mg
		*P* = 41	45.2 ± 7.0	24.1 ± 2.8		1.5–2 mg
Zhang et al. (29) China	Single center	RT = 30	32.60 ± 5.06	23.58 ± 3.48	Hysteroscopy	0.48 mg/kg/h
		RT = 30	31.13 ± 3.95	22.50 ± 2.64		0.6 mg/kg/h
		*P* = 30	32.70 ± 5.25	23.70 ± 2.73		5 mg/kg/h
Zhang et al. (27) China	Single center	RT = 64	33.73 ± 6.27	21.54 ± 3.01	Hysteroscopy	0.2 mg
		*P* = 64	34.58 ± 7.48	21.76 ± 3.03		1.5 mg
		RT + *P* = 65	33.49 ± 6.26	22.29 ± 3.17		0.2 + 0.5 mg
Fan et al. (7) China	Single center	RB = 40	43.95 ± 7.51	22.93 ± 3.02	Hysteroscopy	0.25 mg
		*P* = 43	42.05 ± 9.071	23.03 ± 2.96		2.5 mg
Matsumoto et al. (21) Japan	Multi-center	RB = 30	50.0 ± 15.7	NA	Gynecological Surgery	12 mg/kg/h
		*P* = 30	46.5 ± 16.9			0.4–1 mg/kg/h
Wang et al. (23) China	Single center	RB = 102	40.0 (8.8)	21.9 (20.3, 23.8)	Cervical Conization	0.2 mg
		*P* = 102	40.6 (8.2)	22.6 (20.8, 24.9)		2 mg
Yue et al. (26) China	Single center	RT = 100	31 (19–41)	20.7 (16.9–26.0)	Surgical abortion	0.3 mg
		*P* = 100	30 (20–53)	20.8 (16.4–26.6)		2 mg
Huang et al. (20) China	Single center	RB = 52	50.02 ± 10.82	23.32 ± 3.53	Hysteroscopy	0.2 mg
		RB = 52	47.02 ± 10.61	23.33 ± 3.47		0.25 mg
		RB = 52	47.98 ± 11.78	22.52 ± 2.77		0.3 mg
		*P* = 52	47.73 ± 11.29	23.21 ± 3.29		2 mg
Tan et al. (22) China	Single center	RB + *P* = 125	35.0(29.5–39.0)	20.7 (19.5–22.4)	Hysteroscopy	0.125 + 2.5 mg
		*P* = 125	34.0(31.5–39.0)	21.0 (19.9–21.8)		2.5 mg
Wang et al. (24) China	Single center	RB = 84	31.5 (7.3)	21.6 (2.9)	Gynecological Surgery	0.2–0.3 mg
		*P* = 84	30.5 (6.8)	21.8 (3.3)		1.5-3 mg
Xie et al. (25) China	Single center	RT = 30	70.2 ± 6.3	22.6 ± 4.5	Hysteroscopy	0.2 mg
		*P* = 30	71.8 ± 6.7	23.1 ± 6.2		2 mg
Yang et al. (30) China	Single center	RT = 9	18–40 overall	20–28 overall	Hysteroscopy	0.27 mg
		*P* = 8				2.0 mg
Zhou et al. (28) China	Single center	*P* = 30	34.1 ± 6.4	22.2 ± 3.0	Hysteroscopy	3.5 μg/mL
		RT = 30	33.8 ± 8.2	21.2 ± 2.6		0.05 mg
		RT = 30	36.0 ± 7.8	21.2 ± 2.4		0.1 mg
		RT = 30	33.6 ± 6.8	21.3 ± 2.7		0.15 mg
		RT = 30	33.5 ± 6.6	22.4 ± 2.5		0.2 mg

RB, remimazolam besylate; RT, remimazolam tosylate; P, Propofol; BMI, Body mass index.

**Table 2:** Characteristics of included studies.

Study	Overall AEs	Hypotension	Low SpO2 90%	Movement	Pain at the injection site	Recovery time; Awakening time (m)	Nausea and vomiting	Bradycardia	Operation time	Success rate
Zhang et al. (6)	*R* **=** 6	1	4	15	1	3.31 ± 1.3	NA	0	13.2 ± 4.2	NA
	*P* **=** 60	5	21	20	33	1 ± 0.02		1	12.6 ± 4.7	
Zhang et al. (6)	Ra = 4	NA	1	NA	0	NA	1	NA	12.27 ± 5.94	NA
	Rb = 5		1		0		1		12.23 ± 3.66	
	*P* = 12		4		7		1		11.27 ± 4.68	
Zhang et al. (27)	*R* = 34	6	2	24	0	4.46 ± 0.66	0	0	11.34 ± 4.62	NA
	*P* = 117	27	25	3	39	3.98 ± 0.83	0	2	13.27 ± 6.25	
	*R* + *P* = 16	10	2	2	0	2.83 ± 0.75	0	1	12.91 ± 6.64	
Fan et al. (7)	*R* = 3	NA	NA	NA	NA	10.20 ± 0.34	NA	NA	NA	37
	*P* = 29					7.91 ± 0.47				43
Matsumoto et al. (21)	*R* = 13	NA	NA	NA	NA	NA	9	NA	158 ± 55	NA
	*P* = 16						9		151 ± 46	
Wang et al. (23)	*R* = 183	31	22	33	1	5.0 ± 3.3	6	4	NA	NA
	*P* = 329	51	43	36	36	3.00 ± 2	9	6		
Yue et al. (26)	*R* = 82	0	4	21	2	9.3 ± 8.27	1	0	6.3 ± 6.	81
	*P* = 52	2	4	20	9	8.6 ± 9	0	6	5.6 ± 6	78
Huang et al. (20)	Ra = 44	NA	5	31	0	5.37 ± 1.47	0	NA	16.17 ± 4.13	46
	Rb = 47		6	26	0	6.88 ± 1.62	1		15.94 ± 4.92	49
	Rc = 57		11	16	0	8.06 ± 1.56	2		15.4 ± 4.46	51
	*P* = 76		20	26	16	8.71 ± 1.88	5		15.99 ± 4.65	50
Tan et al. (22)	RP = 73	NA	2	25	0	3.0 ± 1.5	2	0	12.6 ± 5.25	NA
	*P* = 127		12	23	39	3.6 ± 2.25	0	2	12.3 ± 3.75	
Wang et al. (24)	*R* = 42	1	NA	7	3	6.3 ± 1.9	NA	NA	7.0 ± 3	82
	*P* = 89	7		8	38	6.0 ± 1.3			6.8 ± 3.0	84
Xie et al. (25)	*R* = 16	NA	NA	5	4	13.5 ± 4.9	2	NA	NA	29
	*P* = 41			1	21	11.6 ± 4.2	1			30
Yang et al. (30)	*R* = 13	NA	NA	6	0	5.2 ± 2.7	NA	NA	10 ± 4.1	NA
	*P* = 13			2	8	7.6 ± 2.9			9.9 ± 5.8	
Zhou et al. (28)	*P* = 24	10	8	NA	NA	NA	NA	3	NA	NA
	*R* = 15	7	2					4		
	*R* = 11	4	4					2		
	*R* = 13	3	6					1		
	*R* = 11	3	5					2		

R, Remimazolam; P, Propofol; RP, Remimazolam+ Propofol, NA, Not available.

### Risk of Bias assessment

Overall, the quality was moderate to high. Nine studies demonstrated low risk of bias across all domains. Four studies had some concerns regarding the blinding of outcome assessment. This risk of bias was mainly due to the randomization procedure, the allocation procedure, and other aspects. No studies were judged to have a high risk of bias in any domain (Risk of bias outcomes in Supplementary material).

### Primary outcome

#### Adverse events

The total number of overall AEs in the propofol group was higher than the total number of patients, as some of the patients showed more than 1 AEs. So, we compared the two groups by the rate of AEs per patient in both groups. The AEs rate in the propofol group was 985/739 = 1.33, while in the remimazolam group, it was 688/1026 = 0.67. The relative rate of AEs between the two groups was (1.33/0.67 = 1.98), showing a significant difference between the two groups (almost two times) (*p*  0.0001).

### Secondary outcomes

#### Respiratory depression (SpO2  90%)

**Figure 2:**
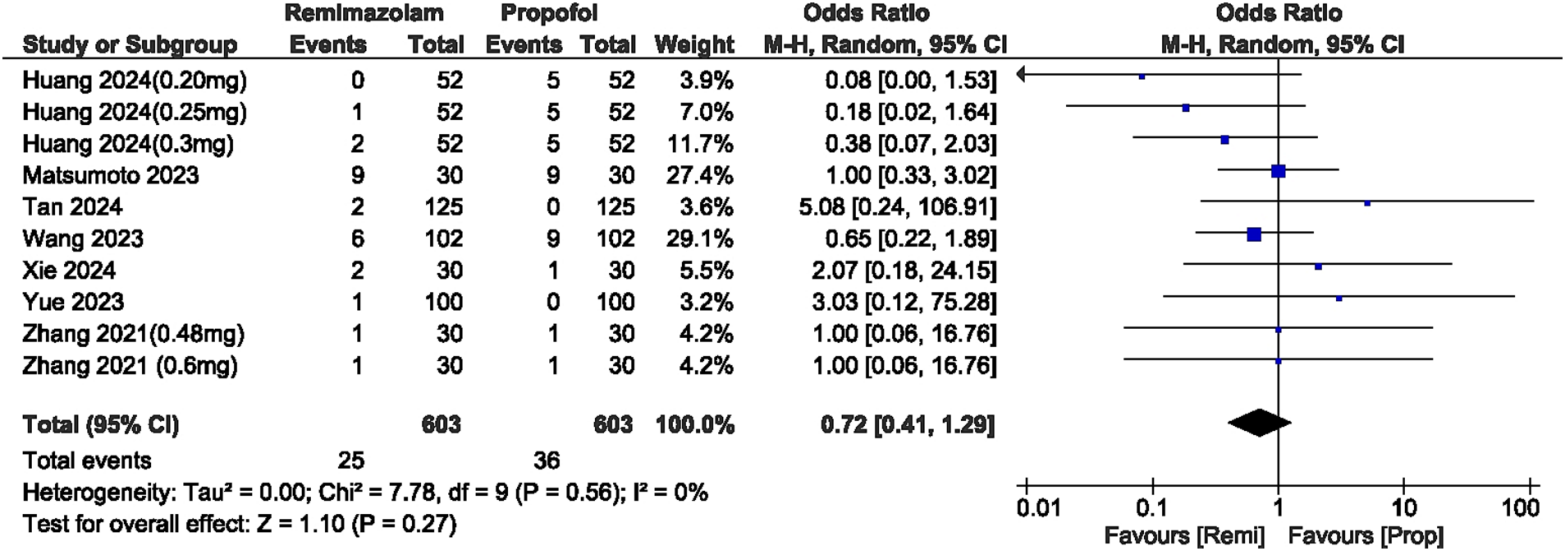
Forest plot of respiratory depression (Low O_2_  90%).

Remimazolam was associated with significantly lower risk of respiratory depression (Low SpO2  90%) compared to propofol (OR, 0.25; 95% CI, 0.17–0.39; *p*  0.00001; *I*^2^ = 42%) with moderate heterogeneity in the results (Figure 2).

### Hypotension

**Figure 3:**
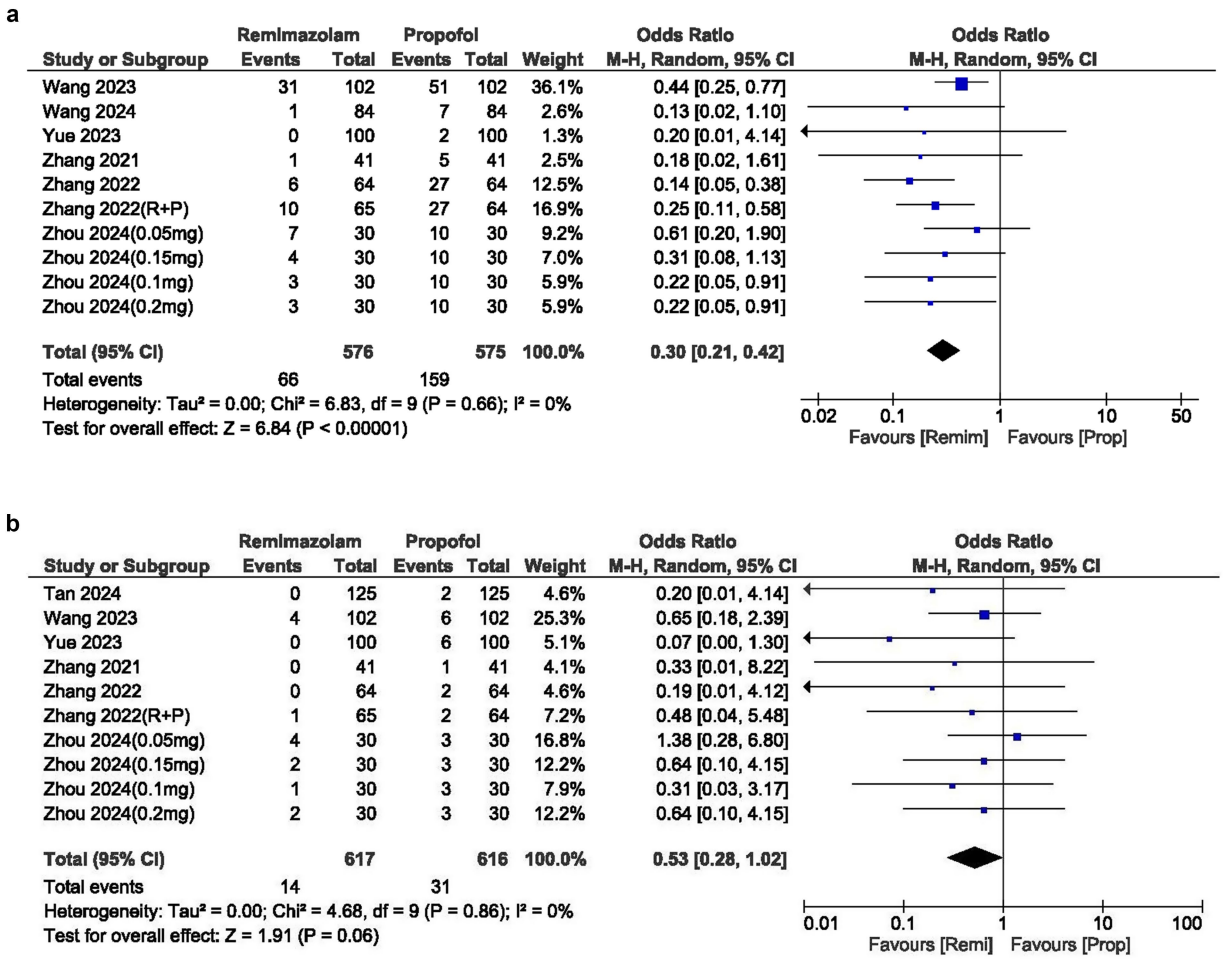
**(a)** Forest plot of hypotension; **(b)** Forest plot of bradycardia.

The incidence of hypotension was significantly lower with remimazolam, yielding an OR of 0.30 (95% CI: 0.21–0.42; *p*  0.00001). The *I*^2^ statistic was 0%, indicating no heterogeneity in the results (Figure 3a).

### Bradycardia

The OR for bradycardia between the two groups was 0.53 (95% CI: 0.28–1.02; *p* = 0.06), indicating no significant difference and showing no heterogeneity (*I*^2^ = 0%). However, the overall incidence of bradycardia was lower in the remimazolam group (Figure 3b).

### Nausea and vomiting

**Figure 4:**
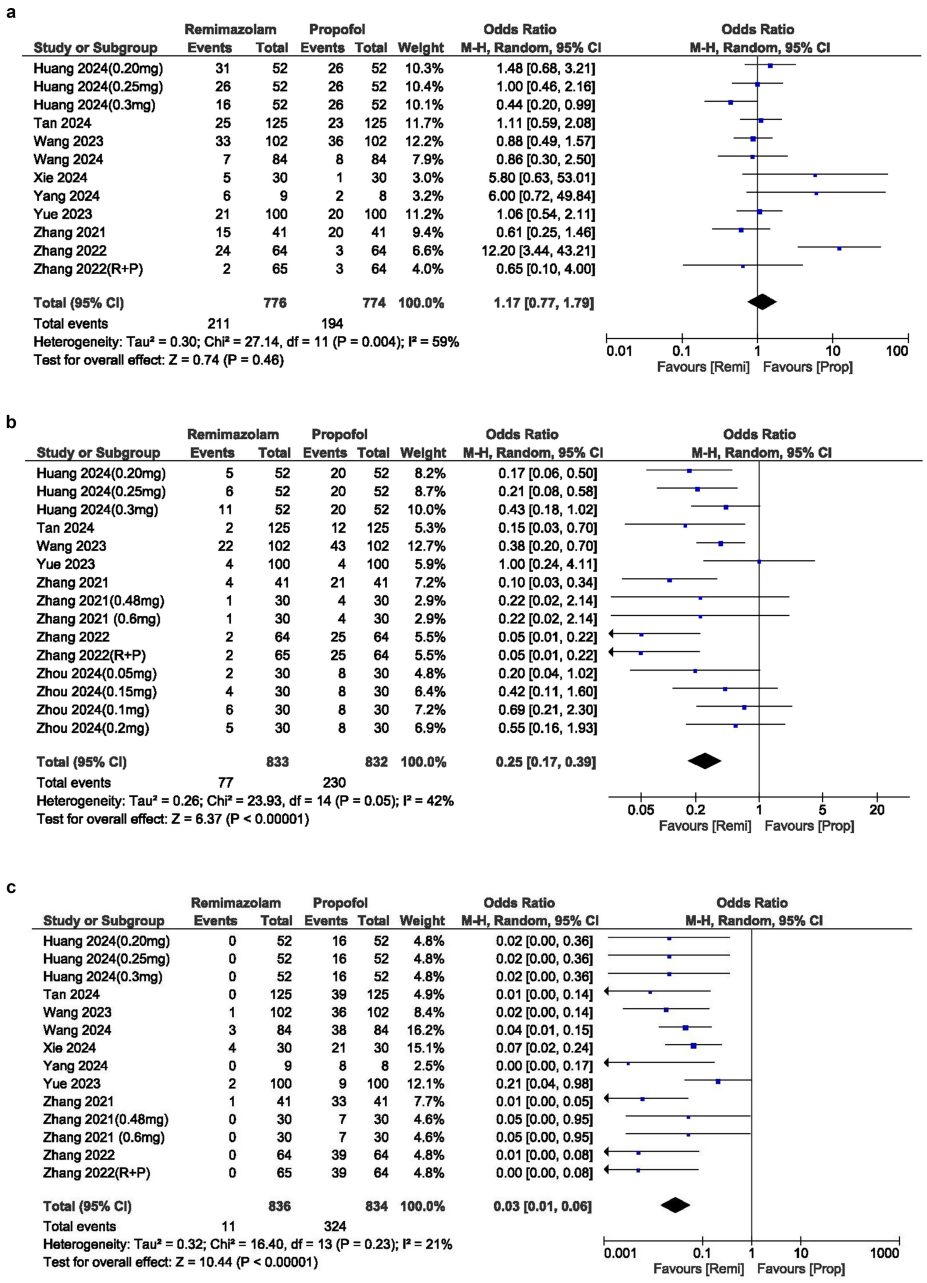
**(a)** Forest plot of hypotension; **(b)** Forest plot of bradycardia; **(c)** Forest plot of movement during procedure.

The cases of nausea and vomiting were similar between the two groups, with an OR of 0.72 (95% CI: 0.41–1.29; *p* = 0.27). The *I*^2^ statistic was 0%, indicating no heterogeneity in the results (Figure 4a).

### Pain

The incidence of injection site pain was significantly lower in the remimazolam group compared to the propofol group, with an OR of 0.03 (95% CI: 0.01–0.06; *p*  0.00001; *I*^2^ = 21%) (Figure 4b).

### Movement during procedure

The cases of movement during the procedure were similar between the two groups, with an OR of 1.17 (95% CI: 0.77–1.79; *p* = 0.46). The *I*^2^ statistic was 59%, indicating moderate heterogeneity in the results (Figure 4c).

### Sedation success

Only five studies reported sedation success with the injected sedative drugs. There was no significant difference in the sedation success rate between the two groups, with an OR of 0.84 (95% CI: 0.48–1.46; *p* = 0.54; *I*^2^ = 0%) (Supplementary Figure S1).

### Recovery parameters

Emergence time was similar between the two groups (MD, 0.18 min; 95% CI, −0.3, 0.65; *I*^2^ = 0%) (Supplementary Figure S2).

### Operation time

Operation time also showed no difference between the two groups (MD, 0.02 min; 95% CI, −1.0, 1.03; *I*^2^ = 99%) with higher heterogeneity (Supplementary Figure S3).

### Sensitivity analyses

Sensitivity analyses that excluded each study one by one did not significantly alter the main findings, indicating the robustness of the results. Additionally, the findings remained consistent in analyses restricted to studies that utilized standardized sedation protocols.

### Publication Bias

Visual inspection of funnel plots showed a little asymmetry, but this was not enough for us to we can conclude the presence of publication bias. Moreover, the Egger’s test also could not find any significant publication bias (*p* = 0.44) (Supplementary Figure S4).

## Discussion

This meta-analysis aimed to evaluate the safety and efficacy of remimazolam compared to propofol in patients undergoing various gynecological procedures. Our findings suggest that remimazolam is associated with a significantly lower risk of overall AEs compared to propofol. Secondary outcomes further highlight the favorable safety profile of remimazolam, including markedly reduced risks of respiratory depression, hypotension, and injection site pain.

Remimazolam exhibits a significant safety advantage compared to propofol, as patients report significantly fewer adverse events, approximately half as many (0.67 versus 1.33 events per patient). This suggests an important 60% reduction in risk and suggests that patients are significantly less prone to experiencing complications. The improved safety profile of remimazolam is linked to its pharmacological characteristics (11). In contrast to propofol, remimazolam is metabolized by tissue esterases in an organ-independent manner, which may lead to more predictable pharmacokinetics. The availability of flumazenil as a specific antagonist offers an additional safety margin that propofol does not provide.

Remimazolam demonstrates significant safety advantages, particularly in two key domains: a 75% reduction in the risk of respiratory depression and a 70% reduction in the risk of hypotension. These complications are common during sedation and often require immediate clinical intervention; thus, their reduction indicates a significant improvement in patient safety. This results in reduced interruptions for clinicians in managing unstable blood pressure or respiratory issues, which improves the procedural process.

Remimazolam also significantly reduces injection site pain, an important negative effect associated with propofol. Patients administered remimazolam exhibited a 97% reduction in the risk of pain during drug administration, thereby converting a typically distressing experience into one of relative comfort. This improvement is particularly crucial for procedures involving active or lightly sedated patients, as pain during injection can increase anxiety and reduce trust in care. Addressing this long-standing issue, remimazolam enhances safety while prioritizing dignity and comfort, benefiting both clinical outcomes and patient-centered care. It was also found that there is no difference in safety outcomes between remimazolam tosylate and besylate formulations. This highlights the importance of essential pharmacologic properties of remimazolam rather than focusing on formulation modifications, which may enhance drug optimization approaches.

Remimazolam provides advantages not only in gynecological contexts but also in other clinical fields such as gastrointestinal endoscopy, surgery, and bronchoscopy (14, 15, 31–33). During procedures like colonoscopies and upper endoscopies, remimazolam showed superior outcomes relative to propofol (31). A significant advantage is its cardiorespiratory stability, which reduces the risk of hypotension and respiratory complications, particularly in elderly patients with comorbidities. Remimazolam’s less severe effect on respiration during bronchoscopy may facilitate smoother procedures and enhance the overall experience for patients undergoing outpatient procedures (34). Furthermore, remimazolam has demonstrated favorable outcomes in multiple surgical procedures, establishing it as a valuable option in clinical practice.

This meta-analysis has multiple strengths that strengthen its credibility. A comprehensive literature search was conducted to include every relevant research study, which reduced bias. The methodology adhered to established guidelines, highlighting significant clinical outcomes such as adverse events and respiratory depression. The majority of the studies included showed high quality and low bias, thereby enhancing the reliability of our findings. The results were consistent across multiple studies conducted in various Asian countries. The strengths identified offer important insights into the safety and efficacy of remimazolam compared to propofol, supporting its application as a preferred anesthetic choice in gynecological procedures.

We should acknowledge the limitations of this meta-analysis. First, all studies were performed in Asia (mostly in China because its commonly used in the East Asia but this is becoming more and more common in all over the world), which raises concerns regarding the generalizability to other populations. The significant heterogeneity observed in the primary outcome, despite being addressed through sensitivity analyses, indicates possible residual confounding due to clinical or methodological diversity, as only a few studies have reported all these (pretreatment, additional sedation, ASA status, BIS score, etc.) characteristics of the included patients. That’s why we were unable to perform subgroup analysis on this basis. Third, the limited sample sizes in specific trials (e.g., *n* = 8 per group) may restrict statistical power. The emphasis on gynecological procedures (e.g., 9/13 studies on hysteroscopy) limits the applicability of findings to wider surgical contexts. Finally, although Egger’s test showed no significant publication bias (*p* = 0.44), minor asymmetry in the funnel plot may suggest a misrepresentation of smaller negative studies.

Our findings suggest that remimazolam may be safer than propofol for sedation during hysteroscopy, especially for patients with a higher risk of respiratory or hemodynamic complications. Further studies should focus on cost-effectiveness, particular patient subgroups (including the elderly and individuals with comorbidities), and optimal dosing strategies. Furthermore, ongoing research investigating cognitive outcomes and patient satisfaction could offer significant findings.

## Conclusion

Remimazolam is a safer alternative to propofol for gynecological sedation, with significantly fewer cases of adverse events, respiratory depression, and hypotension. More large-scale studies are required to verify these findings across various populations and procedural contexts.

## Data Availability

The original contributions presented in the study are included in the article/Supplementary material, further inquiries can be directed to the corresponding author.
