# Supplementary material for: Safety and efficacy of remimazolam versus propofol sedation in gynecological procedures: a meta-analysis of East Asian randomized trials
Source: Front Med (Lausanne). 2025 Nov 26;12:1701785. doi: 10.3389/fmed.2025.1701785 (PMC12689587; doi:10.3389/fmed.2025.1701785)
Supplement: Supplementary file 1 [file Data_Sheet_1.docx]

**Supplementary Material**

**Fig 1** Forest plot of Sedation Success


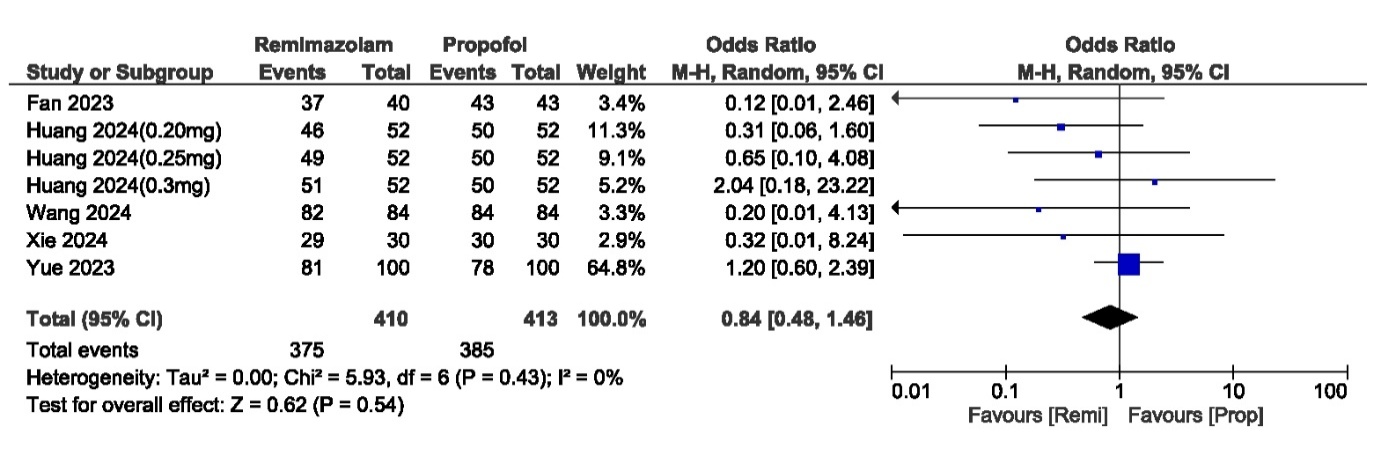


**Fig 2** Forest plot of Recovery Parameters


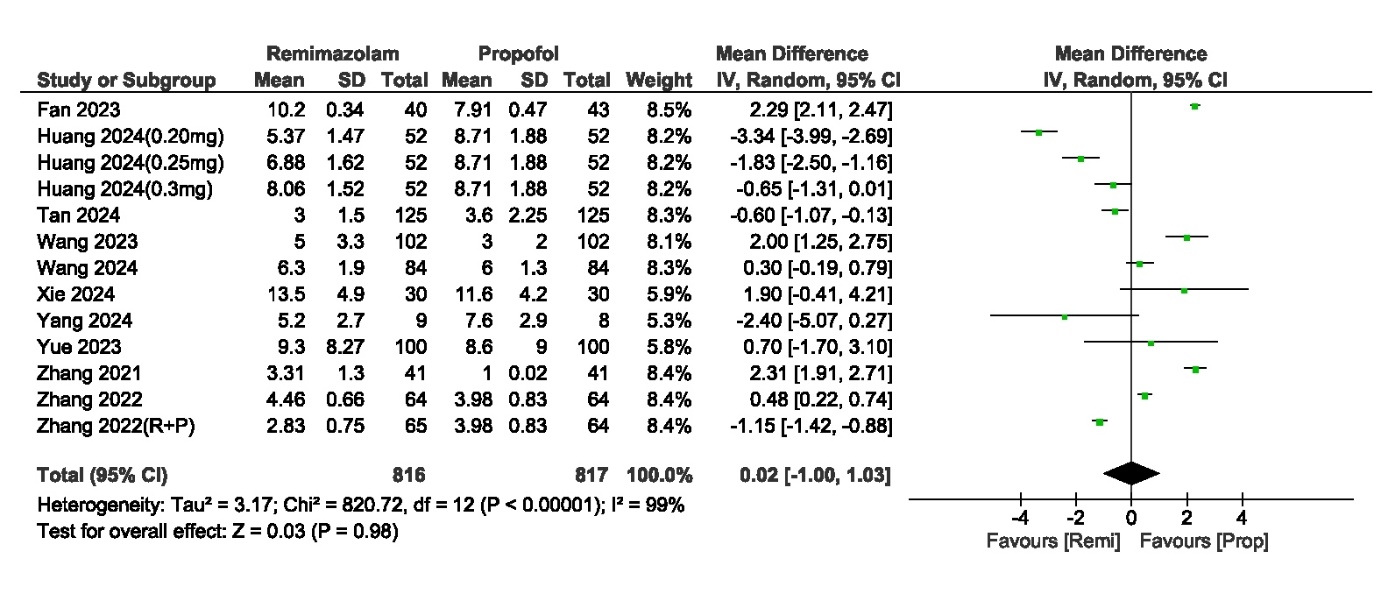


**Fig 3** Forest plot of Operation time


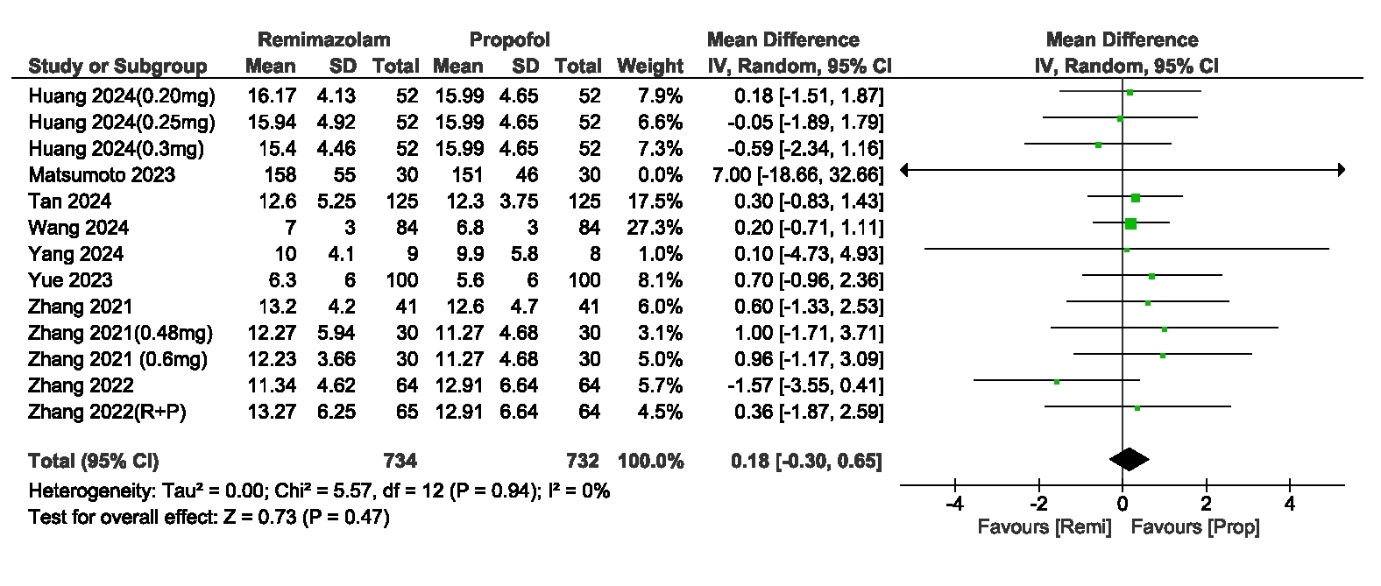


**Fig 4** Funnel plot of adverse events


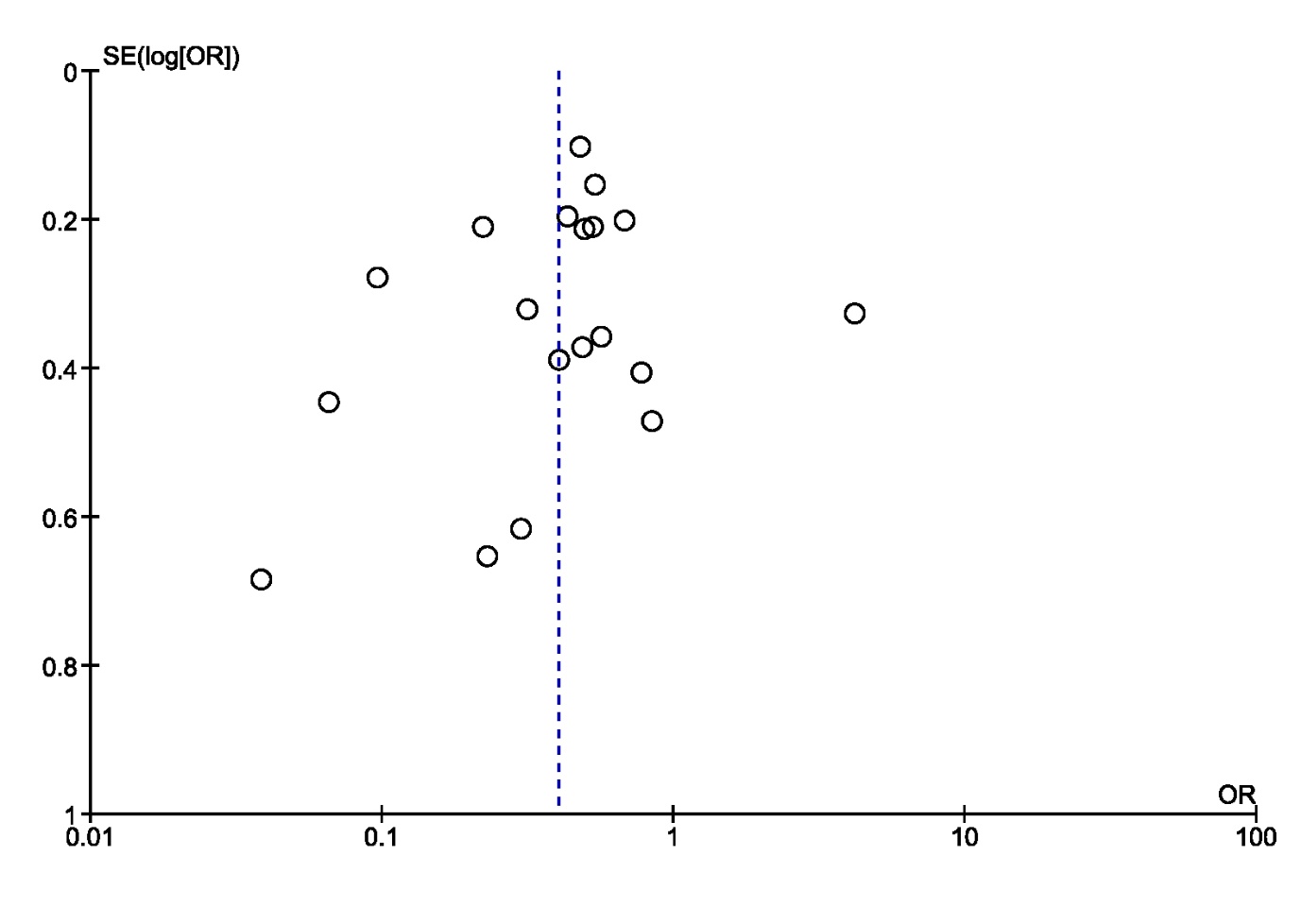


**Risk of bias**

**
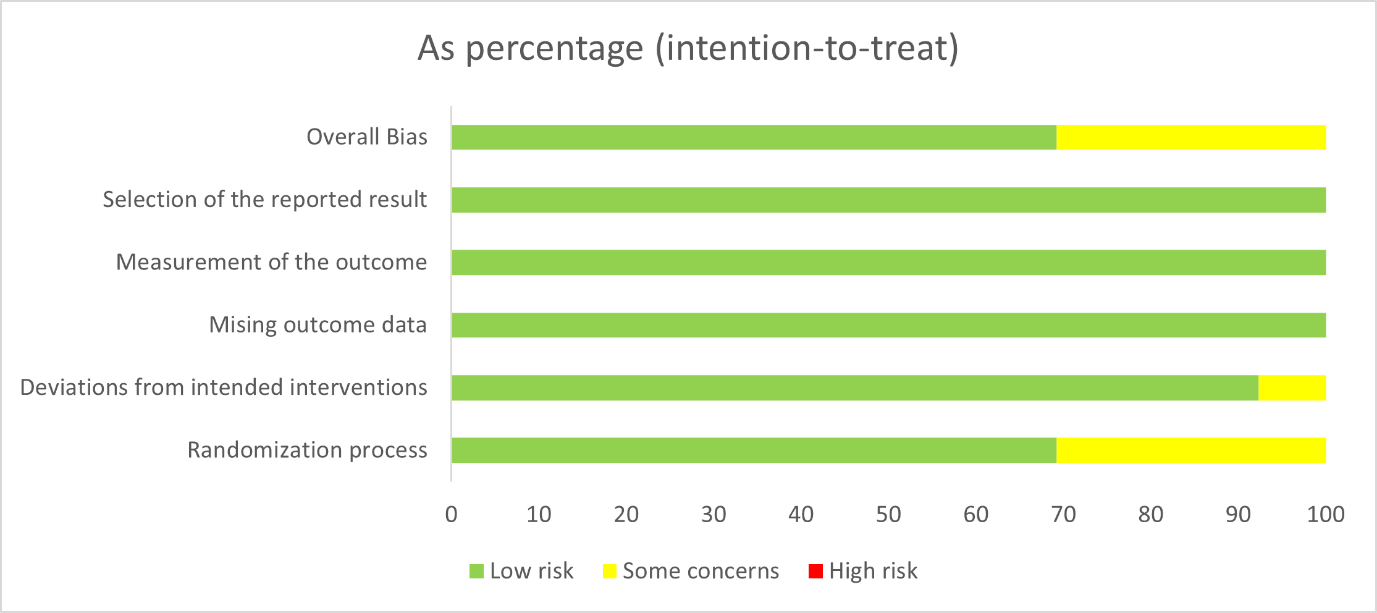
**

**
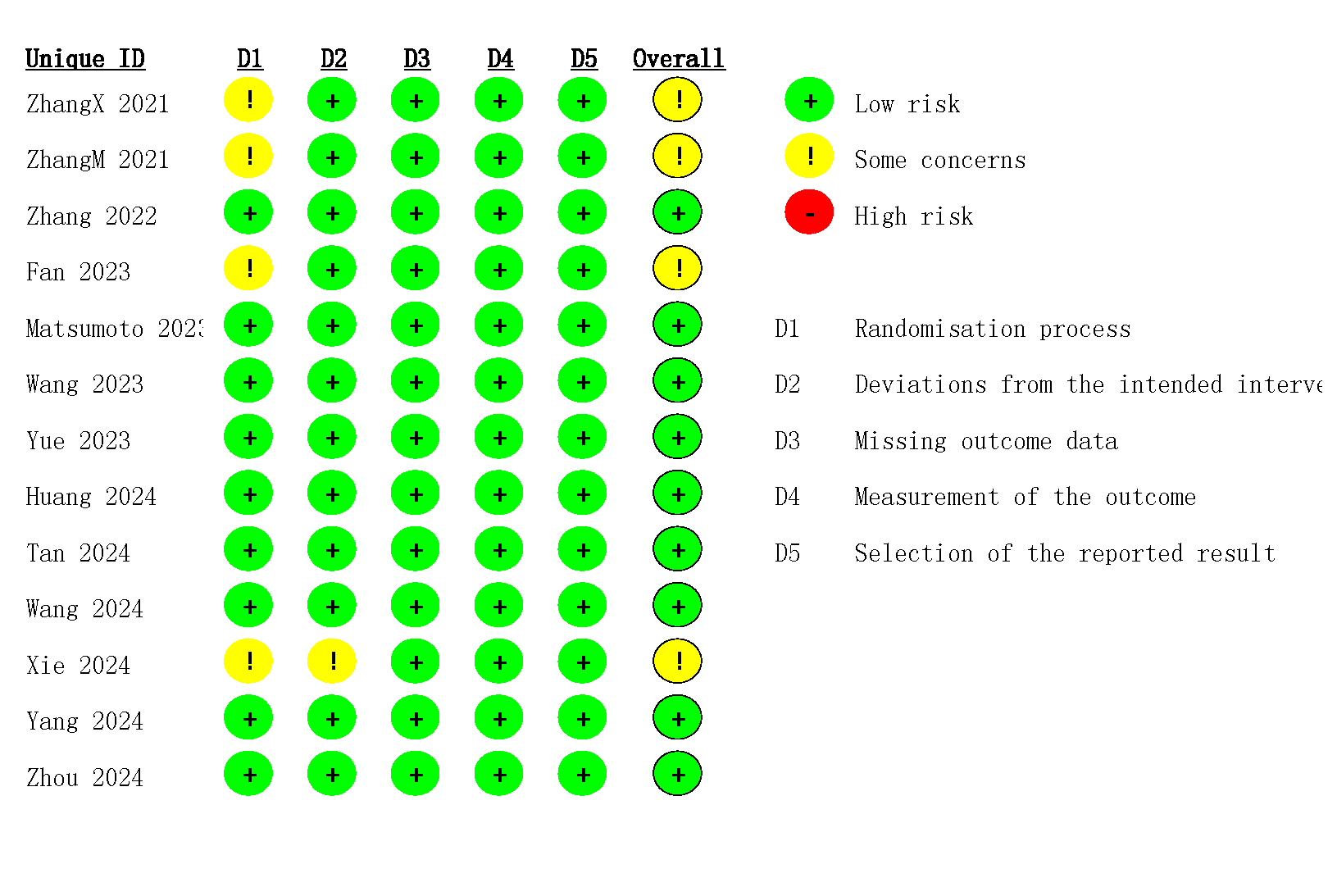
**

**Search strategy**

**PubMed**

1. (("Propofol"[Title/Abstract] OR "Aquafol"[Title/Abstract] OR "Diprivan"[Title/Abstract] OR "Disoprivan"[Title/Abstract] OR "Disoprofol"[Title/Abstract] OR "Fresofol"[Title/Abstract] OR "Ivofol"[Title/Abstract] OR "Propovan"[Title/Abstract] OR "Lipuro"[Title/Abstract])
2. ("Remimazolam"[Title/Abstract] OR "CNS-7056"[Title/Abstract] OR "Byfavo"[Title/Abstract]))
3. ("hysteroscopic"[Title/Abstract] OR "gynecological surgery"[Title/Abstract] OR "hysteroscopy"[Title/Abstract] OR "cervical conization"[Title/Abstract])
4. 1 AND 2 320

EMBASE

('propofol'/exp OR 'propofol') AND ('remimazolam'/exp OR 'remimazolam')
